# Fused multicyclic polyketides with a two-spiro-carbon skeleton from mangrove-derived endophytic fungus *Epicoccum nigrum* SCNU-F0002†

**DOI:** 10.1039/d0ra05532h

**Published:** 2020-08-03

**Authors:** Zhangyuan Yan, Jialin Li, Geting Ye, Tao Chen, Meimei Li, Yanmin Liang, Yuhua Long

**Affiliations:** Guangzhou Key Laboratory of Analytical Chemistry for Biomedicine, School of Chemistry, South China Normal University Guangzhou 510006 China Yuhualong68@hotmail.com

## Abstract

A pair of uncommon fused multicyclic polyketides with a two- spiro-carbon skeleton, (±)-isoepicolactone, (±)-1, and one new isobenzofuranone monomer (4), together with four other known biosynthetically related compounds were isolated from the fermentation of an endophytic fungus, *Epicoccum nigrum* SCNU-F0002, which was isolated from the fresh fruit of the mangrove plant *Acanthus ilicifolius L*. Comprehensive spectroscopic analysis, X-ray crystallography, together with calculated ECD, were employed to define the structures. The antibacterial and COX-2 inhibitory activities of the compounds (1–6) were evaluated. A possible biogenetic pathway of (±)-isoepicolactone was confirmed.

## Abstract 2

A pair of uncommon fused multicyclic polyketides, (±)-isoepicolactone and one new isobenzofuranone monomer, together with four other known biosynthetically related compounds were isolated from the fermentation of fungus *Epicoccum nigrum* SCNU-F0002.

## Graphical abstract

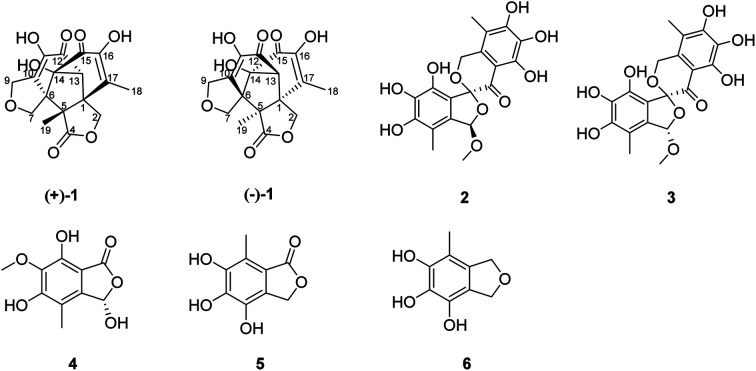

## 1. Introduction

Dimeric natural products possess fascinating molecular architectures and display remarkable bioactivities.^1–4^ So far, various kinds of dimeric derivatives, including polyketides,^5,6^ alkaloids,^7^ terpenoids^8,9^ and peptides,^10^ have been obtained from different species of marine mangrove endophytic fungi and terrestrial organisms. An overview of the molecular structures of dimerics reveals that the biosynthetically originate from the convergence of two monomer, which are formed mostly by intermolecular [4 + 2] cycloaddition (Diels–Alder reaction),^11,12^ aldol condensation reaction,^13^ [3 + 2] cycloaddition,^14,15^ radical coupling^16^ and [5 + 2] cycloaddition.^17^ Among them, dimeric isoepicolactones possessing a pentacyclic ring system based on a decalin moiety, which is bridged by two spiro-connected furan rings to form a symmetrical carbon skeleton with two spiro centers, have been isolated from the fermentation of an endophytic fungus, *Epicoccum nigrum* SCNU-F0002. The intricate polycyclic skeleton of 1 is unique in natural sources, and has been reported in only three literatures up to date.^18–20^ From the pathway analysis, we know that (±)-isoepicolactone *via* intermolecular [5 + 2] cycloaddition reaction complete the cascade and obtains a complex dimmer compound. Our first time characterize isoepicolactone from natural source proved the priediction of Prof. Trauner,^21^ who accomplished the elegant total synthesis of epicolatone using intermolecular [5 + 2] cycloaddition strategy, in that work (±)-isoepenolides are reported as by-products in racemates.

**Fig. 1:**
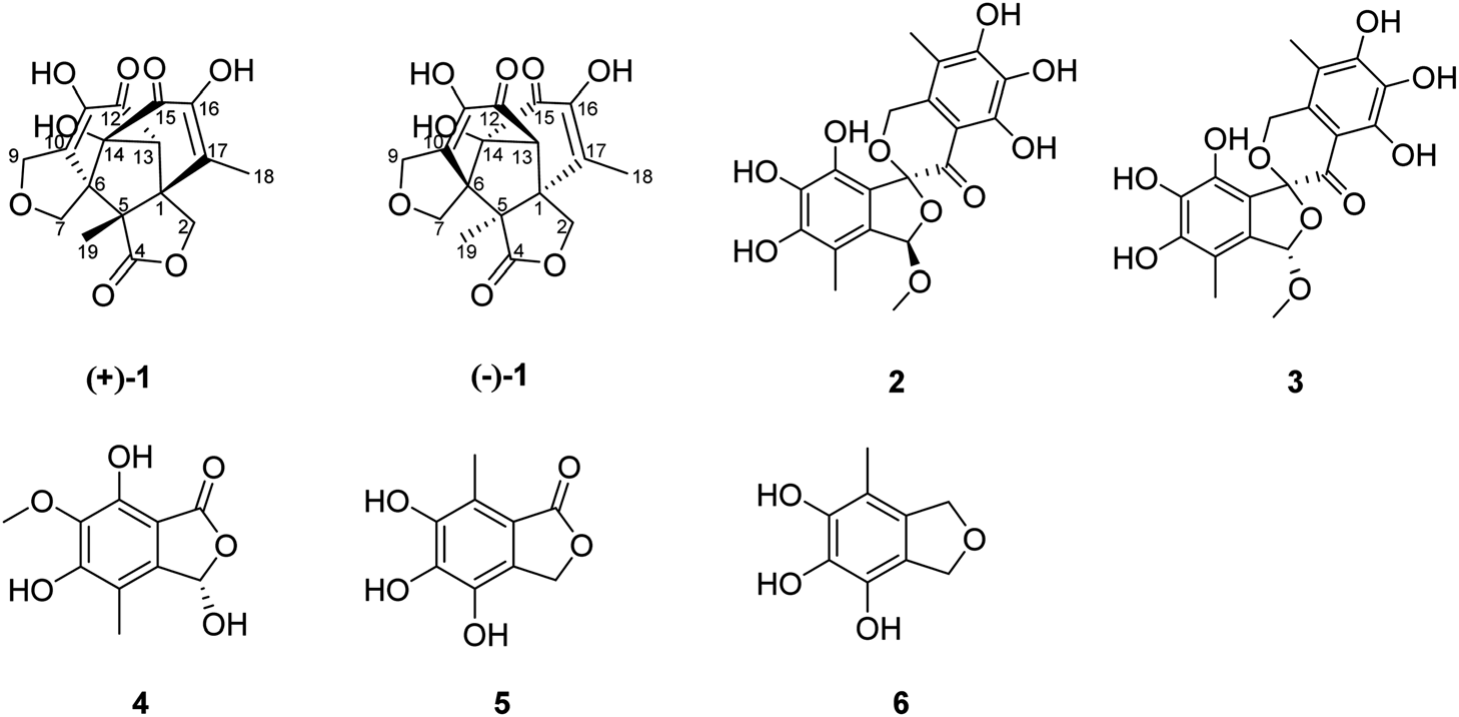
Chemical structures of 1–6.

During our ongoing search for bioactive polyketides derivatives from marine mangrove endophytic fungi, changes in the composition of the culture medium were employed to reinvestigate the secondary metabolites of *Epicoccum nigrum* SCNU-F0002. This led to the isolation of a pair of (±)-isoepicolactone, (±)-1, and one new isobenzofuranone monomer (4), together with four known biosynthetically related compounds (Fig. 1). Herein, the isolation, structural elucidation, biological evaluation, and plausible biosynthetic pathway of (±)-isoepicolactone were described.

## 2. Results and discussion

### 2.1 Structure elucidation

**Fig. 2:**
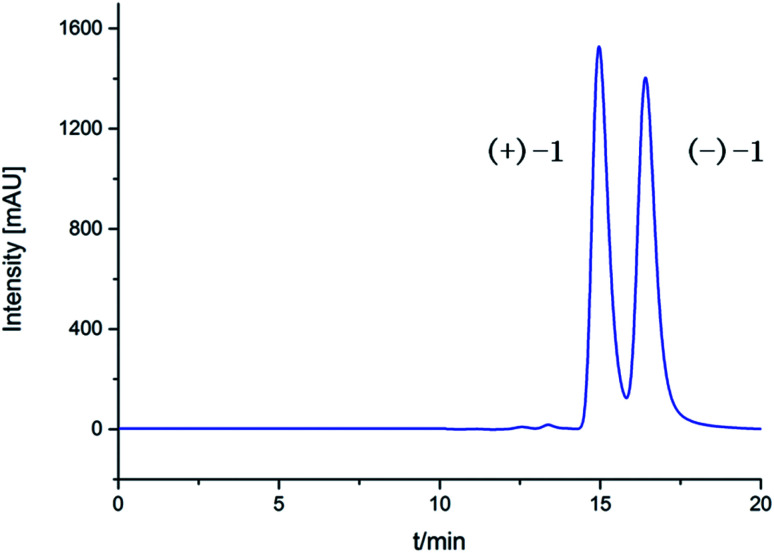
Chiral resolution HPLC spectrum of compounds (±)-1.

**Fig. 3:**
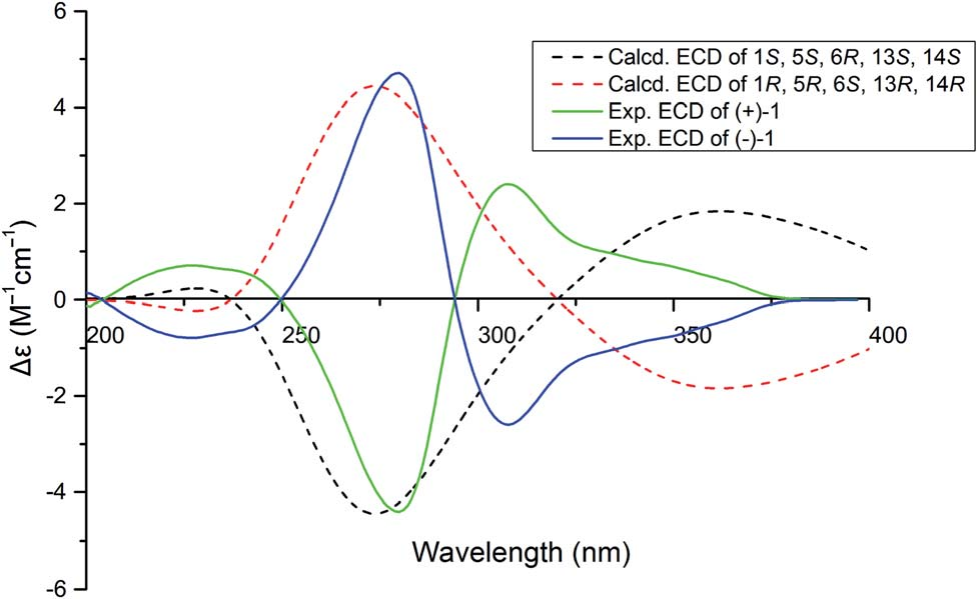
Calculated and experimental ECD spectra of compounds (±)-1.

The EtOAc extract of cultures of *Epicoccum nigrum* SCNU-F0002 was fractionated and purified by repeated silica gel chromatography, chiral-phase HPLC, reversed-phase HPLC, and semipreparative HPLC to afford three new and four known related compounds.

(±)-Iso-epicolactone, (±)-1, was obtained as white solid. The molecular formula was deduced as C_17_H_16_O_18_ based on the HRESIMS at *m*/*z* 347.0775 [M − H]^−^ (calcd for C_17_H_15_O_18_, 347.0772), indicating 10 degrees of unsaturation. IR spectrum exhibited the absorption bands of hydroxyl group (3394 cm^−1^) and carbonyl groups (1757 and 1646 cm^−1^). The ^1^H NMR and HMQC spectra recorded in DMSO gave the signals of three singlets at *δ*_H_ 6.28 (14-OH), 8.84 (16-OH) and 8.97 (11-OH) ppm with no correlations to carbon atoms, which are thus assigned to OH, three oxymethylene protons at *δ*_H_ 4.21 (H-2) and 4.61 (H-2′); 3.74 (H-7) and 3.82 (H-7′); 4.28 (H-9) and 4.38 (H-9′), and two methyls at *δ*_H_ 1.96 (H-18), 1.03 (H-19), one methine at *δ*_H_ 3.08. Analysis of ^13^C NMR, DEPT 135°, and HMQC spectra showed 17 carbon signals that could be assigned as two α,β-unsaturated carbonyls at *δ*_C_ 190.1 (C-12) and 193.3 (C-15), one ester carbonyl at *δ*_C_ 176.5 (C-4), two methyls at *δ*_C_ 13.8 (C-18) and 17.5 (C-19), three methylene signals at *δ*_C_ 66.4 (C-2), 65.6 (C-7) and 67.8 (C-9), one methine carbon at *δ*_C_ 69.1 (C-13), eight quaternary carbons at *δ*_C_ 51.3 (C-1), 60.1 (C-5), 59.6 (C-6), 136.9 (C-10), 140.1 (C-11), 88.4 (C-14), 146.5 (C-16) and 134.7 (C-17). Their structures were identified as isoepicolactone, by analysis of spectroscopic data (1D, 2D NMR) and comparison with literature values (ESI-Table S1†).^21^ The lack of optical activity and cotton effects in the ECD spectrum showed that 1 was racemic. Subsequently, 1 was successfully resolved by HPLC using a chiral ND (2) column, to afford two optically pure enantiomers, (+)-1 and (−)-1, in a ratio of 1 : 1 by chiral-phase HPLC (Fig. 2). The same ECD patterns but opposite cotton effects and the opposite optical rotations of (+)-1 and (−)-1 verified their enantiomeric relationship. In the NOESY spectrum, the correlations from H-13 to H-2′/H-9/H-7′, H-19 to H-2′/H-9/H-7′, H-9 to H-2′revealed the cofacial relationship of these protons, while the cross-peaks of H-18/H-9′ and H-9′/H-2 showed these groups on the opposite side of the molecule. Thereby, the relative configuration has been assigned as 1R*,5R*,6S*,13R*,14R*. By comparing the calculated and experimental ECD spectra (Fig. 3), the absolute configurations of (+)-1 and (−)-1 were determined to be (1*S*,5*S*,6*R*,13*S*,14*S*) and (1*R*,5*R*,6*S*,13*R*,14*R*), respectively (Fig. 1).

**Fig. 4:**
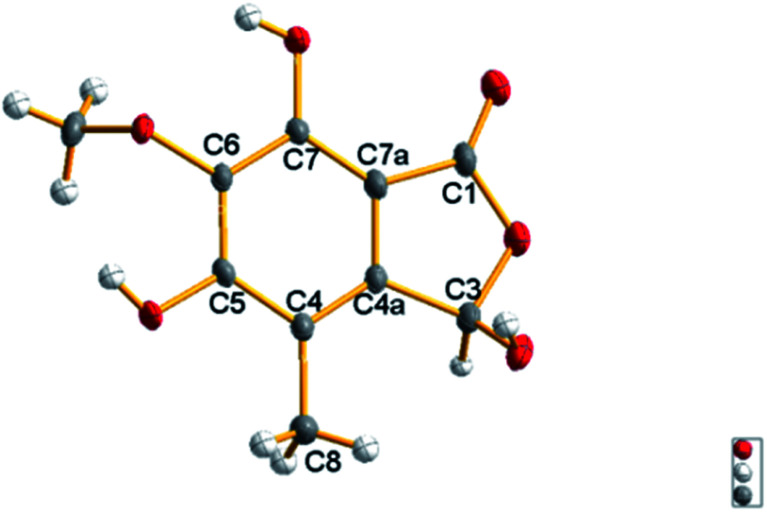
X-ray structure of compound 4.

**Fig. 5:**
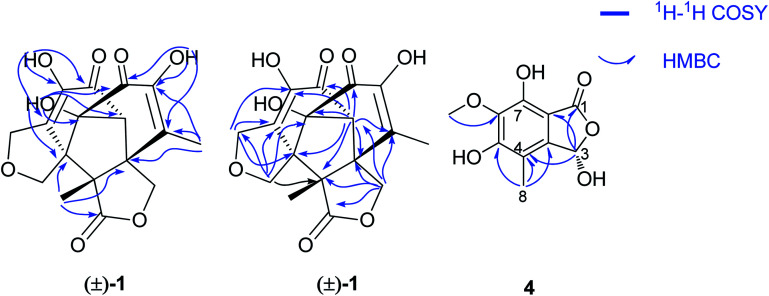
Key HMBC and ^1^H-^1^H COSY correlations of 1 and 4.

The molecular formula of epicoccone F (4) was calculated as C_10_H_10_O_6_ from HRESIMS. The ^13^C NMR data of 4 revealed the presence of 10 carbons, including one lactone carbonyl at *δ*_C_ 171 (C-1), one methoxy carbon at *δ*_C_ 61.2 (6-OMe), one methyl at *δ*_C_ 10.6, one oxymethine at *δ*_C_ 98.5 (C-3), six aromatic carbons at *δ*_C_ 114.3 (C-4), 142.9 (C-4a), 157 (C-5), 148.8 (C-6), 137.6 (C-7) and 104.8 (C-7a). Detailed comparison of their 1D and 2D NMR data from epicoccone D^18^ and F suggested evidence for the same benzofuranone subunit, but compound 4 lacked an oxymethylene proton signal at C-3. The signal was replaced by a hydroxy group (3-OH) in epicoccone F. The HMBC correlations (Fig. 5) from 6-OMe to C-6, and H-8 to C-4/C-4a/C-5 established a methyl group at C-4 and a methoxy group at C-6, which was further supported by HMBC correlations from H-3 to C-1/C-4/C-7a and X-ray single-crystal diffraction analysis (Fig. 4). Finally, all NMR data for 4 were readily assigned by HMBC analysis. Thus, compound 4 was identified as (*S*)-3,5,7-trihydroxy-6-methoxy-4-methylisobenzofuran-1(3*H*)-one (Fig. 1).

The other known compounds were identified as aspermicrone B (2),^22^ aspermicrone C (3),^22^ 4,5,6-trihydroxy-7-methyl phthalide (5),^23^ 4,5,6-trihydroxy-7- methyl-1,3-dihydroisobenzofuran (6)^24^ by comparison of their spectroscopic data with those reported in the literature.

### 2.2 Biological activity

Compounds 1–6 were tested for their antibacterial activities against some of Gram-positive and Gram-negative bacterial strains (*Staphylococcus aureus* ATCC 6538, *Bacillus subtilis* ATCC 6633, *Escherichia coli* ATCC 8739, *Pseudomonas Aeruginosa* ATCC 9027, *Salmonella* ATCC 14028). Unfortunately, they showed no significant activity against the assessed organisms at a dose of 100 μg mL^−1^. Compounds (+)-1 and (−)-1 show weak COX-2 inhibitory activity at 5 μg mL^−1^, inhibition rate 28.8% and 31.2%, the positive control indomethacin inhibition rate 78.9%.

### 2.3 Plausible biogenetic pathway of compounds (±)-1

**Scheme 1:**
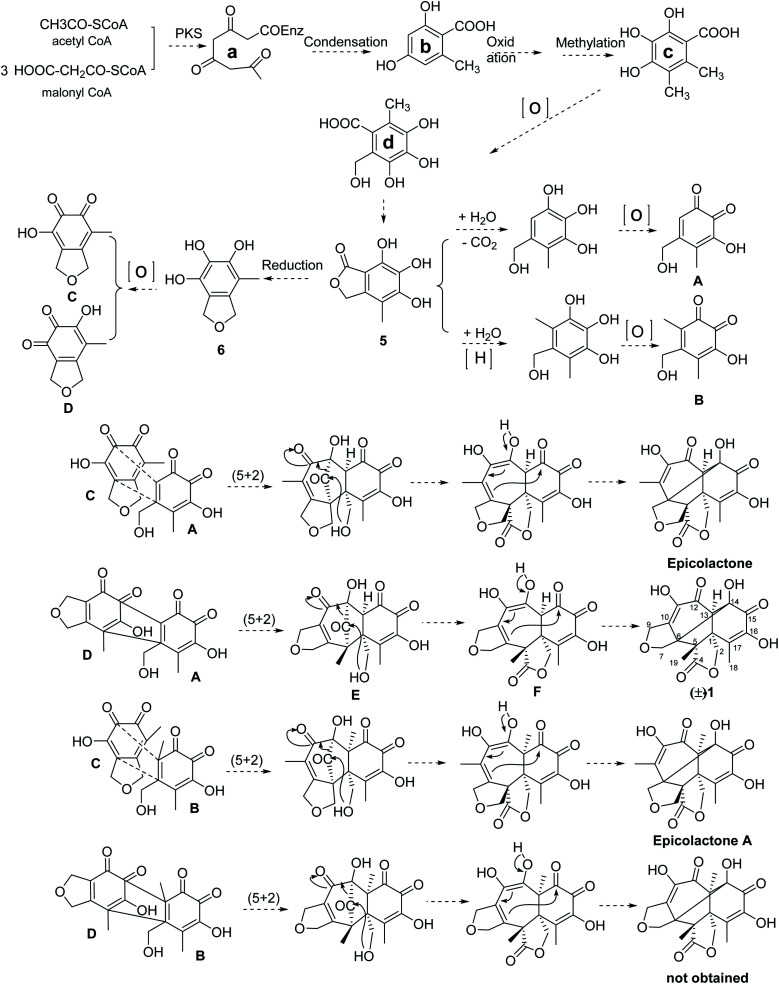
The proposed biosynthesis pathway of (±)-iso-epicolactone (a) epicolactone A^18^ and epicolactone^19,20^ were isolated as previously reported.

The proposed biosynthesis pathway of compounds (±)-1 was performed in Scheme 1. Firstly, the intermediate (a) was formed by three molecules of malony-CoA and one molecular acetyl-CoA based on the HR-PKS. Then, condensation constructed a phenol (b) and the following formed intermediate (c) through the oxidation as well as methylation, which further oxidation to yield intermediate (d). Subsequently, compound 5 could be generated by esterification of intermediate (d) and the following formed compound 6 by reduction. In addition, the intermediate A was formed by the hydrolyzation, decarboxylation and oxidization of compound 5; meanwhile, the intermediate B was obtained by the hydrolyzation, reduction and oxidization of compound 5. The oxidization of epicoccine 6 gave two *o*-quinones intermediates (C and D). Epicolactone derivatives were formed *via* intermolecular [5 + 2] cycloaddition reaction of two *o*-quinones intermediates (A, B, C, D), followed by intramolecular lactone formation. Among them, the [5 + 2] cycloaddition of two *o*-quinones intermediates, A and D, obtained intermediate E, the following intramolecular nucleophilic attack of C_2_-hydroxyl to carbonyl C_4_ formed intermediate F with a lactone moiety, the following tautomerization of C_12_-hydroxyl and intramolecular cyclolization reaction formed C_6_-C_14_ bond, thus the (±)-iso-epicolactone, (±)-1, was formed.

## 3. Experimental section

### 3.1 General experimental procedures

HR-ESI-MS were acquired on a Thermofisher LTQ Orbitrp Elite LC-MS spectrometer (Thermo Fisher Scientific, Inc., Hudson, NH, USA). The ^1^H NMR (600 MHz), ^13^C NMR (150 MHz), and 2D NMR spectra were obtained on a Bruker AVANCE-600 (Bruker BioSpin Corporation, Billerica, MA, USA) using TMS as an internal reference. IR data were measured on a Nicolet 5DX-FTIR (Thermo Fisher Scientific, Inc., Hudson, NH, USA), in KBr discs. Single-crystal data were measured on an Oxford Gemini S Ultra diffractometer (Oxford Instrument, Oxfordshire, UK). Optical rotations were recorded on a Bellingham-Stanley ADP 440 + polarimeter at 25 °C. UV data were recored on a Shimadzu UV-240 spectrophotometer (Shimadzu, Kyoto, Japan). CD data were measured on a J-810 spectropolarimeter (JASCO, Tokyo, Japan). TLC analysis was carried out on silica get plates (Marine chemical Ltd, Qingdao, China). The chiral HPLC separation was accomplished over an Chiral ND (2) column (column size: 250 × 4.6 mm 5 μm; FLM Scientific Instrument Co., Ltd). Phenomenex Luna (Phenomenex, Torrance, CA, USA) C18 column (250 × 10 mm, 5 μm, 5 mL min^−1^) was used for semipreparative HPLC. Silica gel (200–300 mesh, Marine Chemical Ltd, Qingdao, China), and Sephadex LH-20 (GE Healthcare Bio-Sciences AB, Stockholm, Sweden) were used for column chromatography (CC).

### 3.2 Fungal material

The fungal strain SCNU-F0002 was isolated from the fresh fruit of the mangrove plant *Acanthus ilicifolius L* collected from the Qi'ao island Mangrove Nature Reserve in Guangdong province, China. The fungus was obtained using the standard protocol for isolation.^25^ Fungal identification was carried out using a molecular biological protocol by DNA amplification and sequencing of the ITS region.^26^ The sequence data obtained from the fungal strain have been deposited at GenBank with accession no. MN096740. A BLAST result revealed 100% match with the sequence of *Epicoccum nigrum* (compared to MH484012.1). A voucher strain was deposited in School of Chemistry and Environment, South China Normal University, Guangzhou, China, with the access code, SCNU-F0002.

### 3.3 Fermentation, extraction and isolation

The fungus was cultured on autoclaved potato glucose water medium (250.0 g L^−1^ of potato, 3.0 g L^−1^ of artificial sea salts, 20.0 g L^−1^ of glucose) at room temperature under static conditions and daylight for 35 days. Following incubation, the whole liquid medium was filtered through cheesecloth to separate the liquid and mycelia. The former was extracted with EtOAc, while the latter was extracted with MeOH. The MeOH extract was evaporated under reduced pressure to afford an aqueous solution and then extracted with EtOAc. The two EtOAc extracts were combined and concentrated under reduced pressure to give an organic extract (80.0 g). The extract was isolated by column chromatography over silica gel eluting with a gradient of petroleum ether: ethyl acetate from 1 : 0 to 0 : 1 to afford five fractions (fractions 1–5). Fraction 2 (110.0 mg) was applied to Sephadex LH-20 CC and eluted with CH_2_Cl_2_/MeOH (1 : 1) to obtain 11.2 mg of compounds (±)-1 (8.0 mg). Racemic (±)-1 was separated into a pair of enantiomers (+)-1 (1.5 mg, *t*_R_ = 14.9 min) and (−)-1 (1.0 mg, *t*_R_ = 16.4 min) through chiral HPLC (*n*-hexane/isopropanol = 95 : 5, v/v, flow rate: 5.0 mL min^−1^). Fraction 3 (12.0 mg) was purified by semipreparative RP-HPLC (70% acetonitrile/H_2_O) to yield compounds 2 (5.2 mg, *t*_R_ = 8.5 min) and 3 (6.3 mg, *t*_R_ = 11.0 min). Fraction 4 (220.0 mg) was applied to column chromatography over silica gel, eluting with CH_2_Cl_2_/MeOH (200 : 1) and then further purified by Sephadex LH-20 CC eluted with CH_2_Cl_2_/MeOH (1 : 1) to compound 5 (15.8 mg) and compound 6 (4.0 mg). Fraction 5 (40.0 mg) was applied to Sephadex LH-20 CC and eluted with CH_2_Cl_2_/MeOH (1 : 1) to obtain compound 4 (11.2 mg).

### 3.4 Physio–chemical properties of compounds 1and 4

#### 3.4.1 (+)-Iso-epicolactone, (+)-1

White solid; [*α*]_D_^25^ +165.8 (c 0.1, MeOH); −2.5 UV (MeOH) *λ*_max_ (log *ε*): 302 (3.35) nm; IR (KBr) *ν*_max_: 3105, 3058, 1750, 1580, 1450, 1132, 980, 820 cm^−1^; HR-ESI-MS *m*/*z* 347.0775 [M − H]^−^(calcd for 347.0772); ^1^H and ^13^C NMR data, see Table 1.

**Table 1:** ^1^H (600 MHz) and ^13^C NMR (150MHz) data of compounds 1 and 4

Position	1^a^		4^b^	
	*δ*_H_ (*J* in Hz)	*δ*_C_, type	*δ*_H_ (*J* in Hz)	*δ*_C_, type
1		51.3, C		171.0, C
2	4.21, d, (*J* = 10.1);	66.4, CH_2_		
	4.61, d, (*J* = 10.1)			
3			6.44, s	98.5, CH
4		176.5, C		114.3, C
4a				142.9, C
5		60.1, C		157.0, C
6		59.6, C		148.8, C
6-OCH_3_			3.79, s	61.2, CH_3_
7	3.74, d, (*J* = 10.0);	65.6, CH_2_	137.6, C	
	3.82, d, (*J* = 10.0)			
7a				104.8, C
8			2.15, s	10.6, CH_3_
9	4.28, d, (*J* = 16.1);	67.8, CH_2_		
	4.38, d, (*J* = 16.1)			
10		136.9, C		
11		140.1, C		
11-OH	8.97, s	—		
12		190.1, C		
13	3.08, s	69.1, CH		
14		88.4, C		
14-OH	6.27, s	—		
15		193.3, C		
16		146.5, C		
16-OH	8.84, s	—		
17		134.7, C		
18	1.96, s	13.8, CH_3_		
19	1.03, s	17.5, CH_3_		

a Recorded in DMSO-*d*_6_.

b Recorded in MeOH. *J* in Hz. *δ* in ppm.

#### 3.4.2 (−)-Iso-epicolactone, (−)-1

White solid; [*α*]_D_^25^ −196.4 (c 0.1, MeOH); −2.5 UV (MeOH) *λ*_max_ (log *ε*): 302 (3.35) nm; IR (KBr) *ν*_max_: 3105, 3058, 1750, 1580, 1450, 1132, 980, 820 cm^−1^; HR-ESI-MS *m*/*z* 347.0775 [M − H]^−^(calcd for 347.0772); ^1^H and ^13^C NMR data, see Table 1.

#### 3.4.3 Epicoccone D (4)

White solid; [*α*]_D_^25^ +3.2 (c 0.10, MeOH); UV (MeOH) *λ*_max_ (log *ε*): 313 (4.58) nm; IR (KBr) *ν*_max_: 3134, 3033, 2920, 1750, 1450, 1145, 1040, 1060, 960, 820, 780, 762 cm^−1^; HRESIMS *m*/*z* 249.0371 [M + Na]^+^(calcd for 249.0370); ^1^H and ^13^C NMR data, see Table 1.

### 3.5 X-ray crystallographic analysis

#### 3.5.1 X-ray crystal structure analysis of compounds 4

Crystals of 4 was obtained from MeOH–H_2_O (9 : 1, v/v). Crystal X-ray diffraction data were collected on an Agilent Gemini Ultra diffractometer with Cu kα radiation (*λ* = 1.54184 Å). The structure was solved by direct methods (SHELXS-97) and refined using full-matrix least-squares difference Fourier techniques. All non-hydrogen atoms were refined anisotropically. Hydrogen atoms were placed in the ideal geometrical positions and refined isotropically with a riding model. Crystallographic data of 4 have been deposited with the Cambridge Crystallographic Data Centre.

#### 3.5.2 Crystal data of 4

C_10_H_10_O_6_, *M*_r_ = 226.18, triclinic, *a* = 7.7136 (4) Å, *b* = 8.6961 (5) Å, *c* = 9.1169 (5) Å, *α* = 68.073 (5)°, *β* = 79.306 (4)°, *γ* = 64.143 (5)°, *V* = 510.32 (6) Å^3^, space group *P*1̄, *Z* = 2, *D*_c_ = 1.589 g cm^−3^, *μ* = 1.189 mm^−1^, and *F* (000) = 256.0. Crystal dimensions: 0.2 × 0.15 × 0.12 mm^3^. Independent reflections: 1991 (*R*_int_ = 0.0329). The final *R*_1_ values were 0.0463, w*R*_2_ = 0.1371 (*I* > 2*σ*(*I*)). The goodness of fit on F^2^ was 1.037. CCDC number: 1919077.

### 3.6 Antimicrobial activity assay

The antimicrobial activities against five bacterial (*S. aureus* (ATCC 6538), *B. subtilis* (ATCC 6633), *E. coli* (ATCC 8739), *P. aeruginosa* (ATCC 9027) were evaluated in 96-well microtiter plates using a modification of the broth microdilution method.^27,28^ The antibacterial activity test procedure is as previously described.^29^

### 3.7 COX-2 inhibitory activity

The *in vitro* inhibitory of test compounds were evaluated using COX-2 (human) inhibitor screening assay kit (Item no. 701080) supplied by Cayman Chemicals USA. Indomethacin was used as a positive control. The inhibitory activity of the compounds was measured at each concentrations so as to calculating the IC_50_. The procedure for COX-2 in inhibitory immunoassay are as previously reported.^30,31^

### 3.8 ECD calculations

Conformational searches were carried out by means of the Spartan'14 software using Molecular Merck force field (MMFF). All density functional theory (DFT) and time-dependent (TD)-DFT calculations were performed with Gaussian 09 program. Conformers within a 10 kcal mol^−1^ energy window were generated and optimized by DFT calculations at the B3LYP/6-31+G (d, p) level. Conformers with a Bolzmann distribution over 3% were chose for ECD calculations by TD-DFT method at the B3LYP/6-31+G (d, p) level.^32^ The polarizable continuum model for MeOH was used. The calculated ECD curves were generated using the SpecDis 3.0 (University of Würzburg) and Origin Pro 8.5 (Origin Lab, Ltd) from dipole-length rotational strengths by applying Gaussian band shapes with sigma = 0.30 eV.

## 4. Conclusions

Chemical study of *Epicoccum nigrum* SCNU-F0002 collected from the Qi'ao island Mangrove Nature Reserve led to the isolation and identification of three novel compounds, (±)-isoepicolactone, (±)-1, and isobenzofuranone monomer (4). Compounds (+)-1 and (−)-1 showed weak COX-2 inhibitory activity at 5 μg mL^−1^, inhibition rate 28.8% and 31.2%, the positive control indomethacin inhibition rate 78.9%. All compounds revealed weak or no antibacterial activity at a concentration of 100 μg mL^−1^. By exploring the culture conditions, we found that the yield of secondary metabolites in potato glucose water medium was lower than that in rice solid medium. The yield of dimer type compounds was significantly higher than that of rice solid medium studied before.^18^

## Conflicts of interest

No potential conflict of interest was reported by the authors.

## Supplementary Material

RA-010-D0RA05532H-s001

RA-010-D0RA05532H-s002
