# Supplementary material for: Fused multicyclic polyketides with a two-spiro-carbon skeleton from mangrove-derived endophytic fungus Epicoccum nigrum SCNU-F0002
Source: RSC Adv. 2020 Aug 3;10(48):28560–6. doi: 10.1039/d0ra05532h (PMC9055859; doi:10.1039/d0ra05532h)
Supplement: RA-010-D0RA05532H-s001 [file RA-010-D0RA05532H-s001.pdf]

Electronic Supplementary Material (ESI) for RSC Advances.
This journal is © The Royal Society of Chemistry 2020

Fused Multicyclic Polyketides with a Two-Spiro-Carbon Skeleton from Mangrove-Derived Endophytic Fungus *Epicoccum nigrum* SCNU-F0002

Zhangyuan Yan[a], Jialin Li[a], Geting Ye[a], Tao Chen[a], Meimei Li[a], Yanming Liang[a], Yuhua Long[a, *]

[a] Z. Yan, J. Lin, G. Ye, T. Chen, M. Li,Y. Liang, Prof.Y. Long
Guangzhou Key Laboratory of Analytical Chemistry for Biomedicine,
School of Chemistry,
South China Normal University,
Guangzhou 510006, China.

*Corresponding authors. E-mail addresses: Yuhualong68@hotmail.com (Prof.Yuhua Long)

Contents

Fig.S1. HR-ESI-MS spectrum of (±**1**) 3

Fig.S2. ^{1}H- NMR spectrum (DMSO, 600MHz) of (±**1**) 3

Fig.S3. DEPT 135 and ^{13}C-NMR spectrum (DMSO, 600MHz) of compound (±**1**) 4

Fig.S4.^{1}H-^{1}H COSY spectrum (DMSO, 600MHz) of compound (±**1**) 4

Fig.S5. HSQC spectrum (DMSO, 600MHz) of compound (±**1**) 5

Fig.S6. HMBC spectrum (DMSO, 600MHz) of compound (±**1**) 5

Fig.S7. NOESY spectrum of (DMSO, 600MHz) compound (±**1**) 6

Fig.S8. UV spectrum of **(+)-1** in MeOH 6

Fig.S9. Experimental ECD spectrum of **(+)-1** 7

Fig.S11. Experimental ECD spectrum of **(-)-1** 8

Fig.S12. IR spectrum of compound (±**1**) 8

Fig.S13. HR-ESI-MS spectrum of compound **(2)** 9

Fig.S14. ^{1}H- NMR spectrum (CD_3OD, 600MHz) of compound **(4)** 9

Fig.S15. DEPT 135 and ^{13}C-NMR spectrum (CD_3OD, 150MHz) of compound **(4)** .. 10

Fig.S16. ^{1}H-^{1}H COSY spectrum (CD_3OD, 600MHz) of compound **(4)** 10

Fig.S17. HSQC spectrum (CD_3OD, 600MHz) of compound **(4)** 11

Fig.S18. HMBC spectrum (CD_3OD, 600MHz) of compound **(2)** 11

Table S1.deviations in NMR data between natural and synthetic[1] samples of 1 12

Table S2. ECD Computational Result. 12

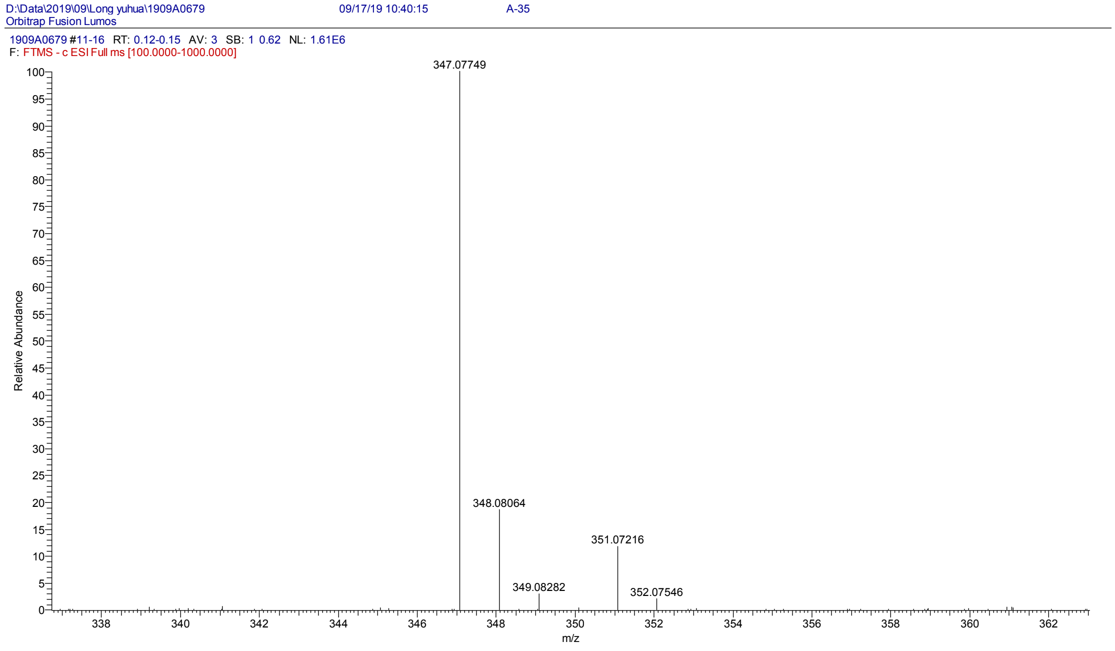

Fig.S1. HR-ESI-MS spectrum of (±**1**)

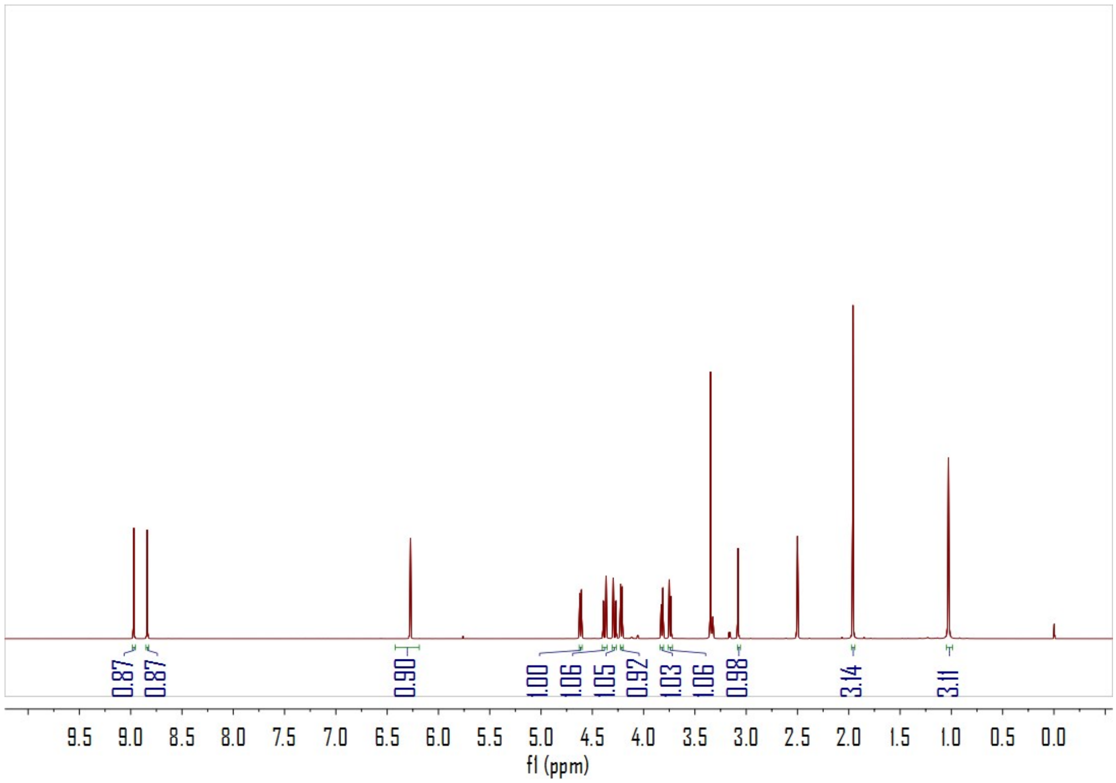

Fig.S2. ^{1}H- NMR spectrum (DMSO, 600MHz) of (±**1**)

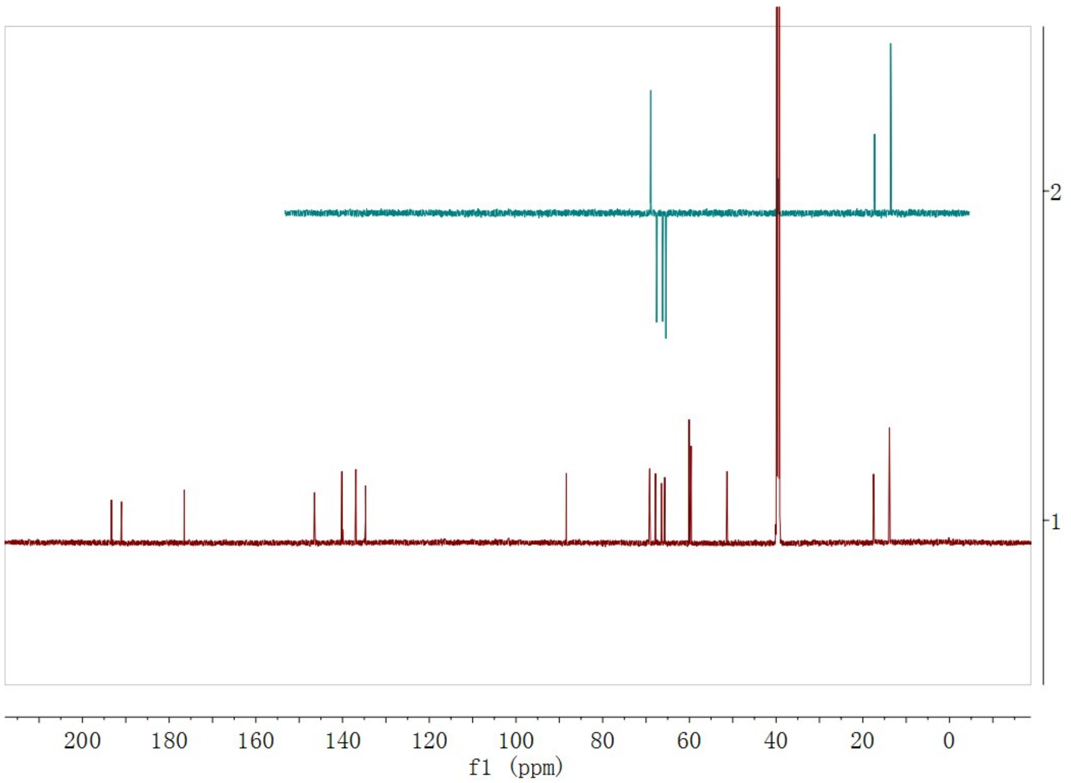

Fig.S3. DEPT 135 and ^{13}C-NMR spectrum (DMSO, 600MHz) of compound (±**1**)

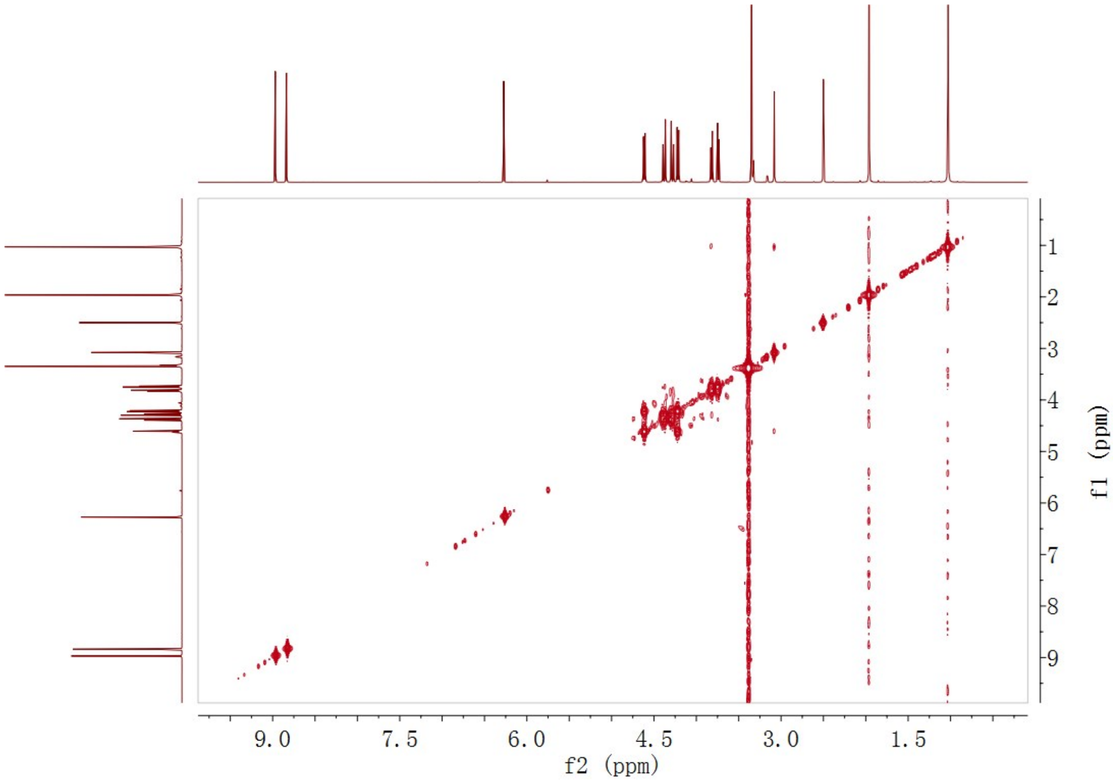

Fig.S4.^{1}H-^{1}H COSY spectrum (DMSO, 600MHz) of compound (±**1**)

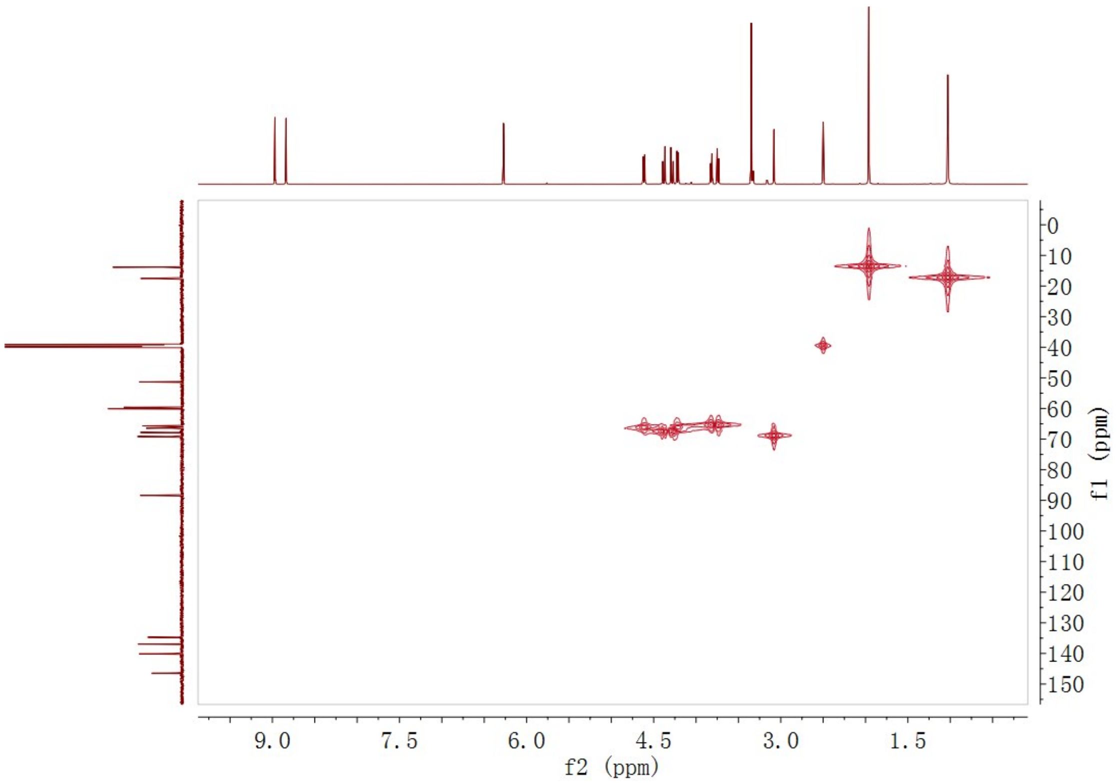

Fig.S5. HSQC spectrum (DMSO, 600MHz) of compound (±**1**)

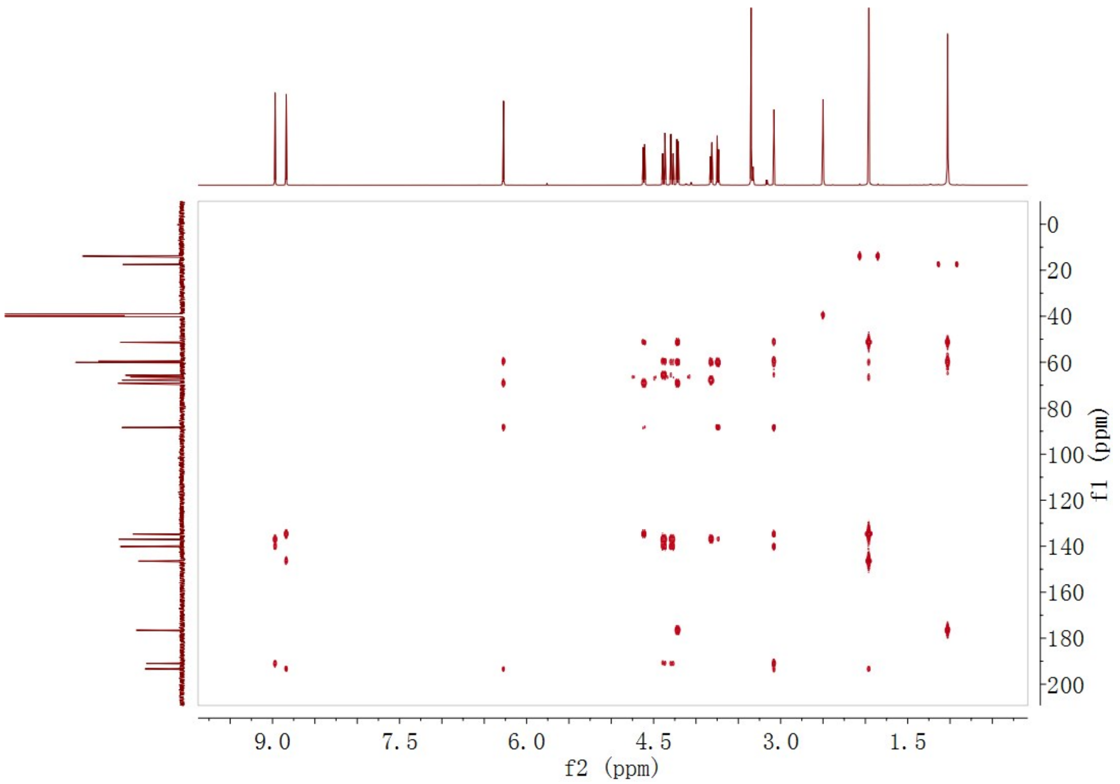

Fig.S6. HMBC spectrum (DMSO, 600MHz) of compound (±**1**)

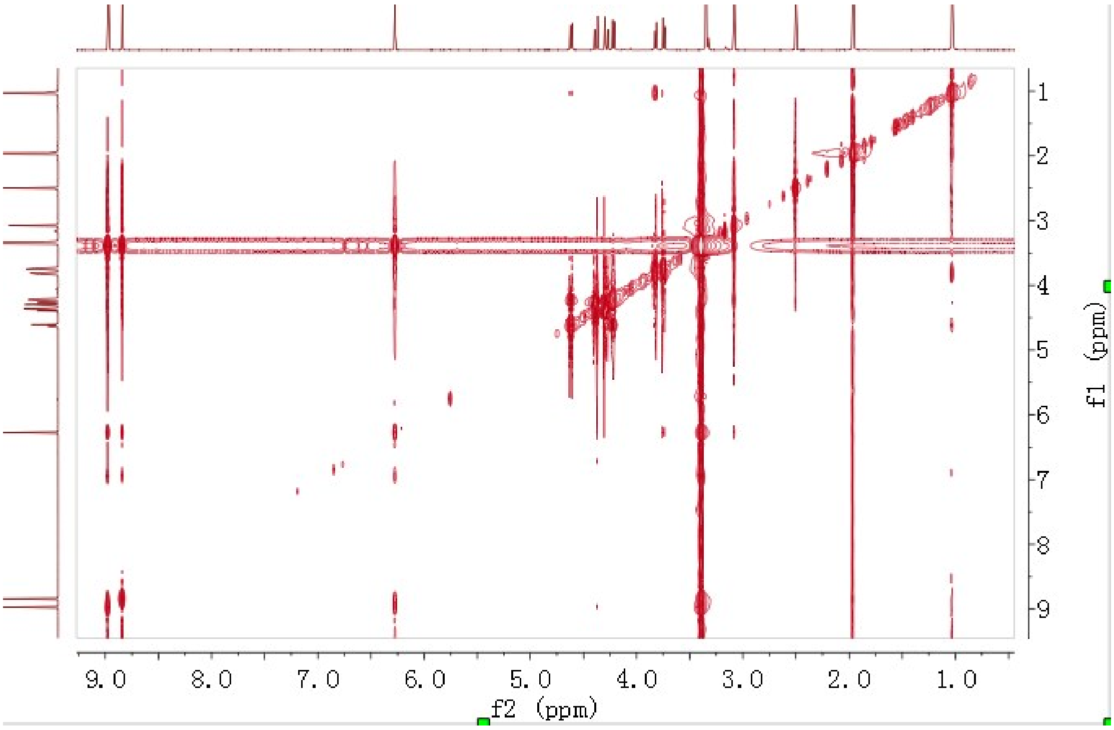

Fig.S7. NOESY spectrum of (DMSO, 600MHz) compound (±**1**)

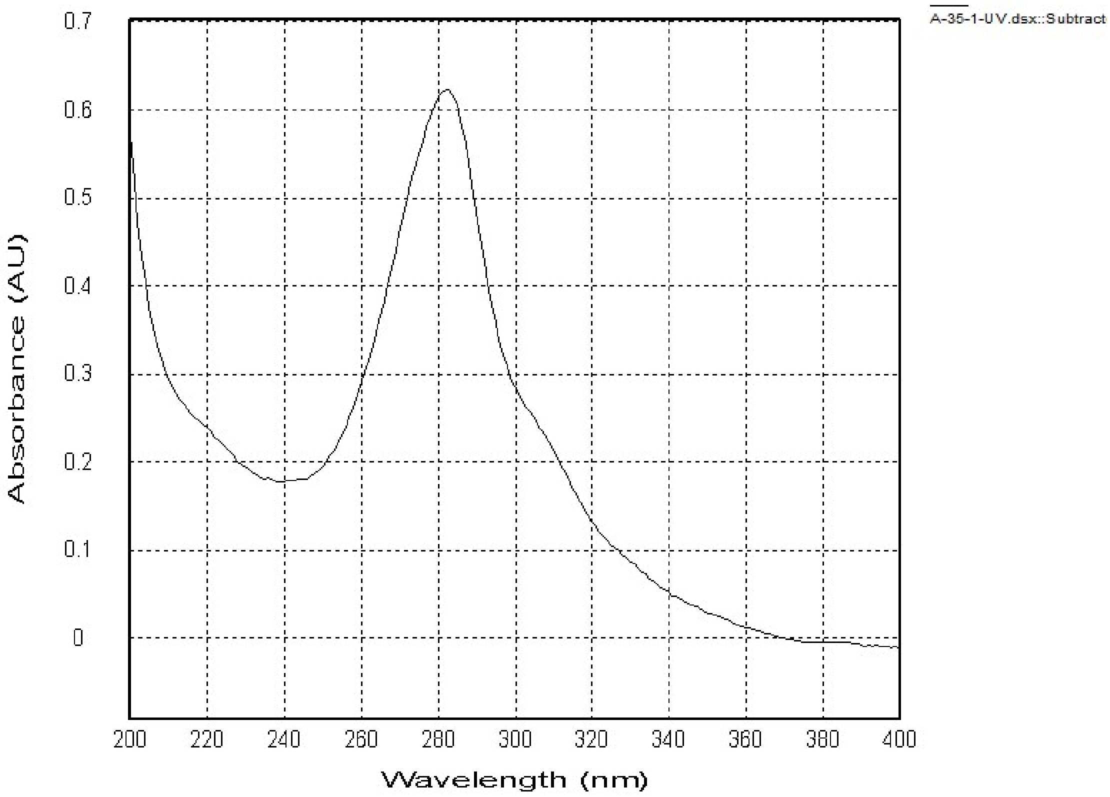

Fig.S8. UV spectrum of **(+)-1** in MeOH

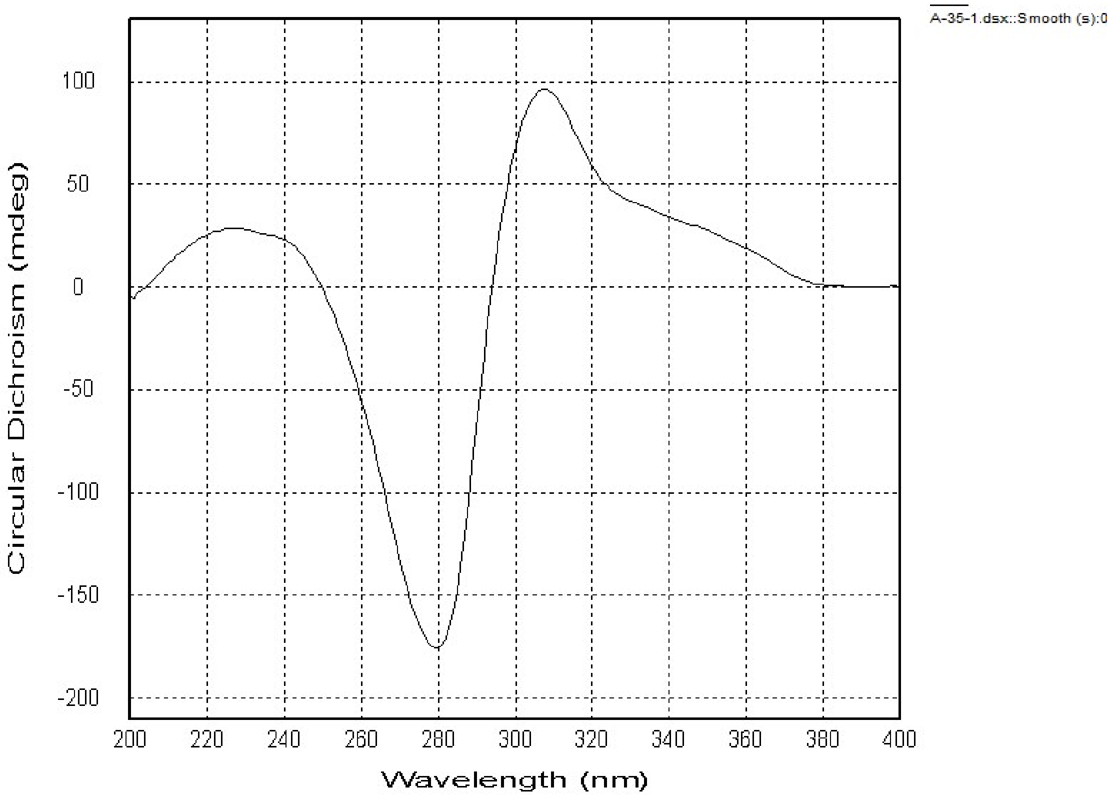

Fig.S9. Experimental ECD spectrum of **(+)-1**

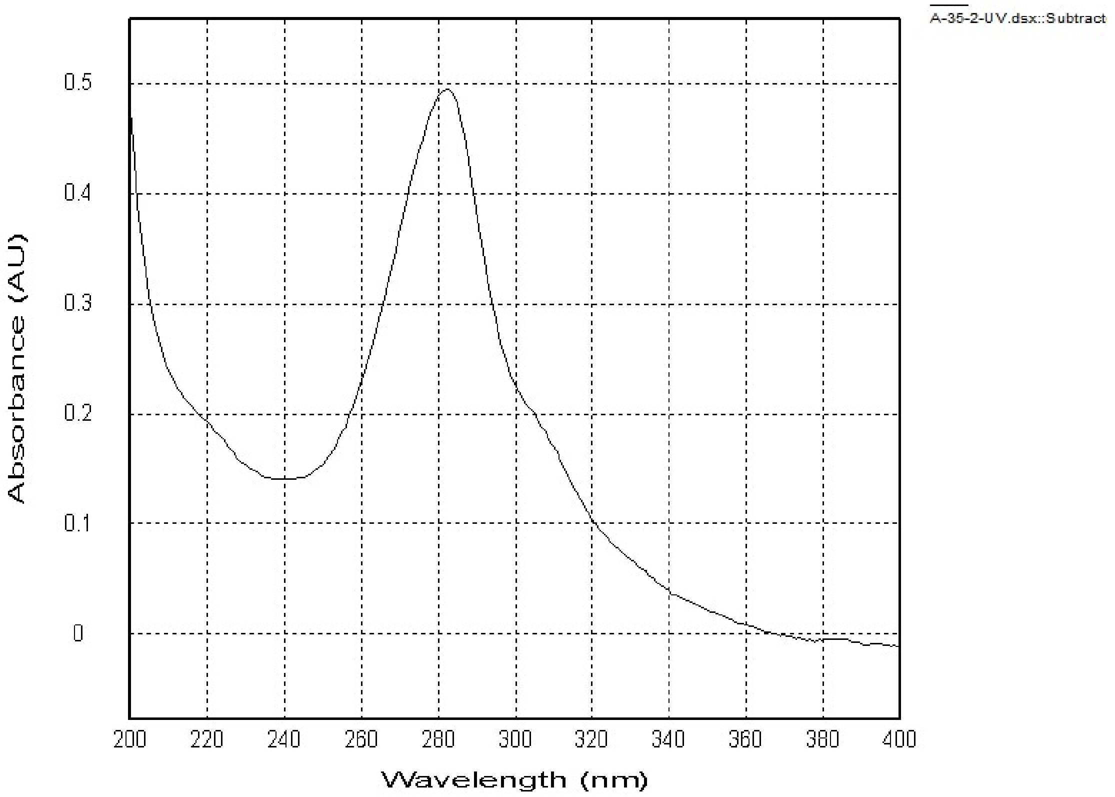

Fig.S10. UV spectrum of **(-)-1** in MeOH

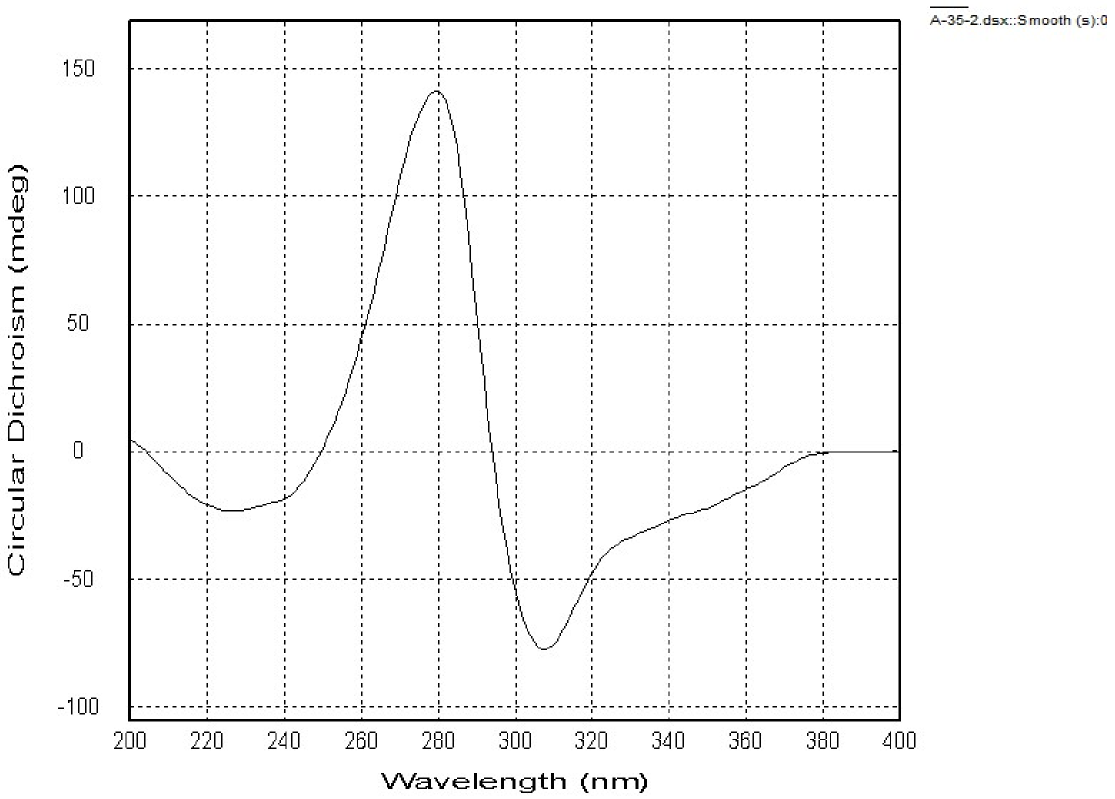

Fig.S11. Experimental ECD spectrum of **(-)-1**

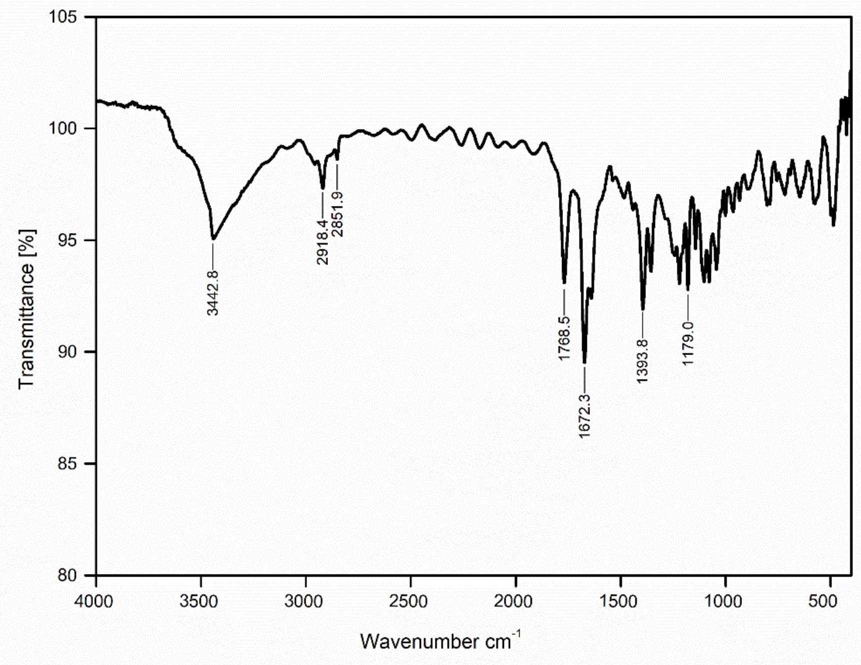

Fig.S12. IR spectrum of compound (±**1**)

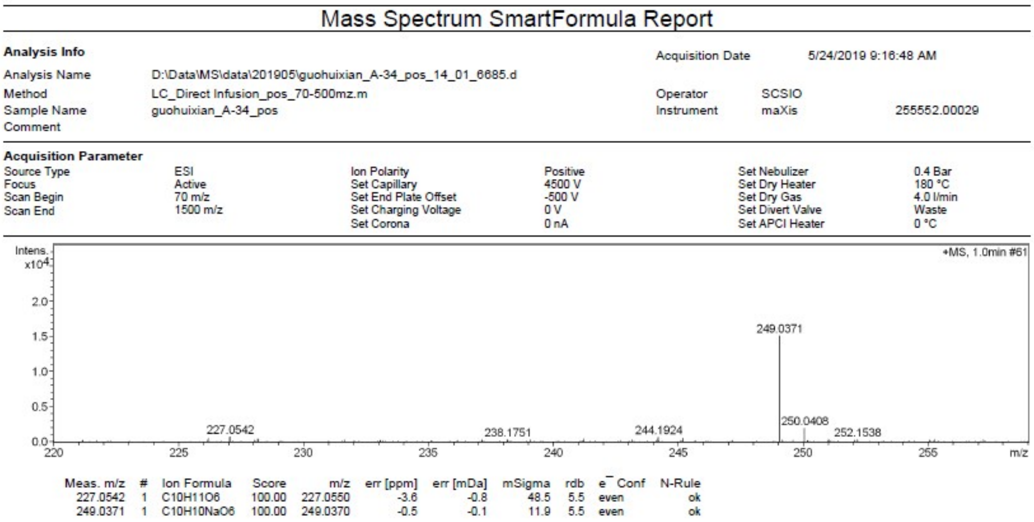

Meas. m/z	#	Ion Formula	Score	m/z	err [ppm]	err [mDa]	mSigma	rdb	e¯ Conf	N-Rule
227.0542	1	C10H11O6	100.00	227.0550	-3.6	-0.8	48.5	5.5	even	ok
249.0371	1	C10H10NaO6	100.00	249.0370	-0.5	-0.1	11.9	5.5	even	ok

Fig.S13. HR-ESI-MS spectrum of compound **(2)**

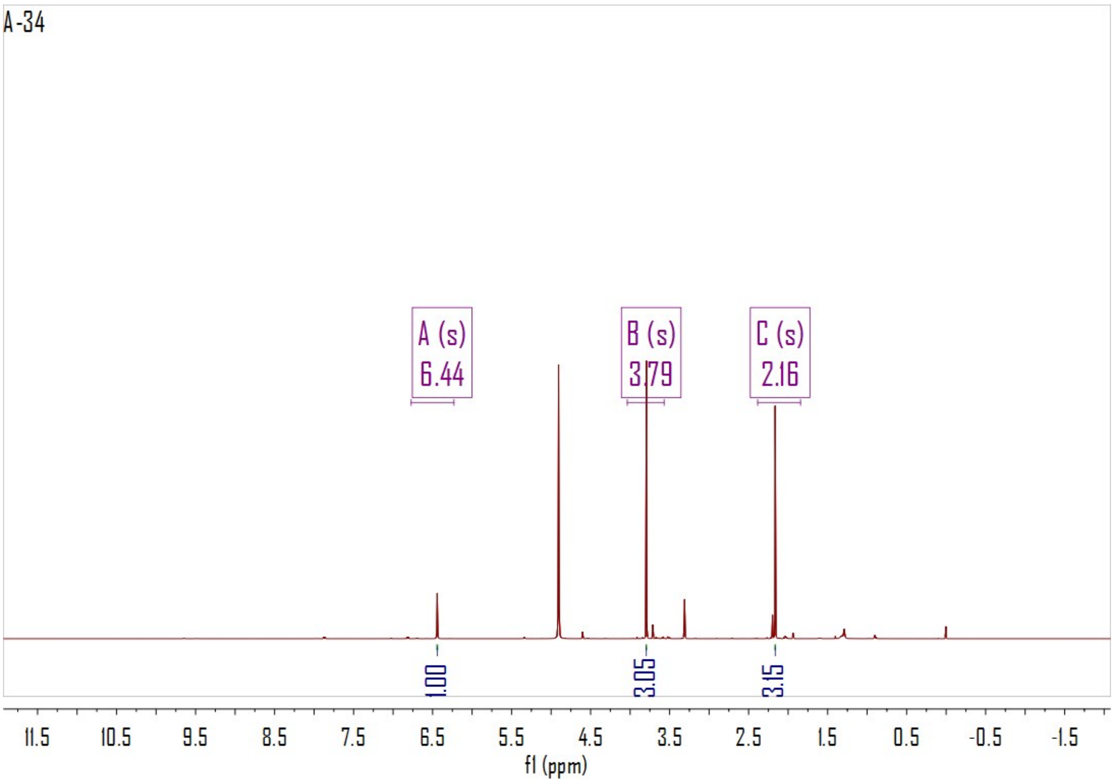

Fig.S14. ^{1}H- NMR spectrum (CD_3OD, 600MHz) of compound **(4)**

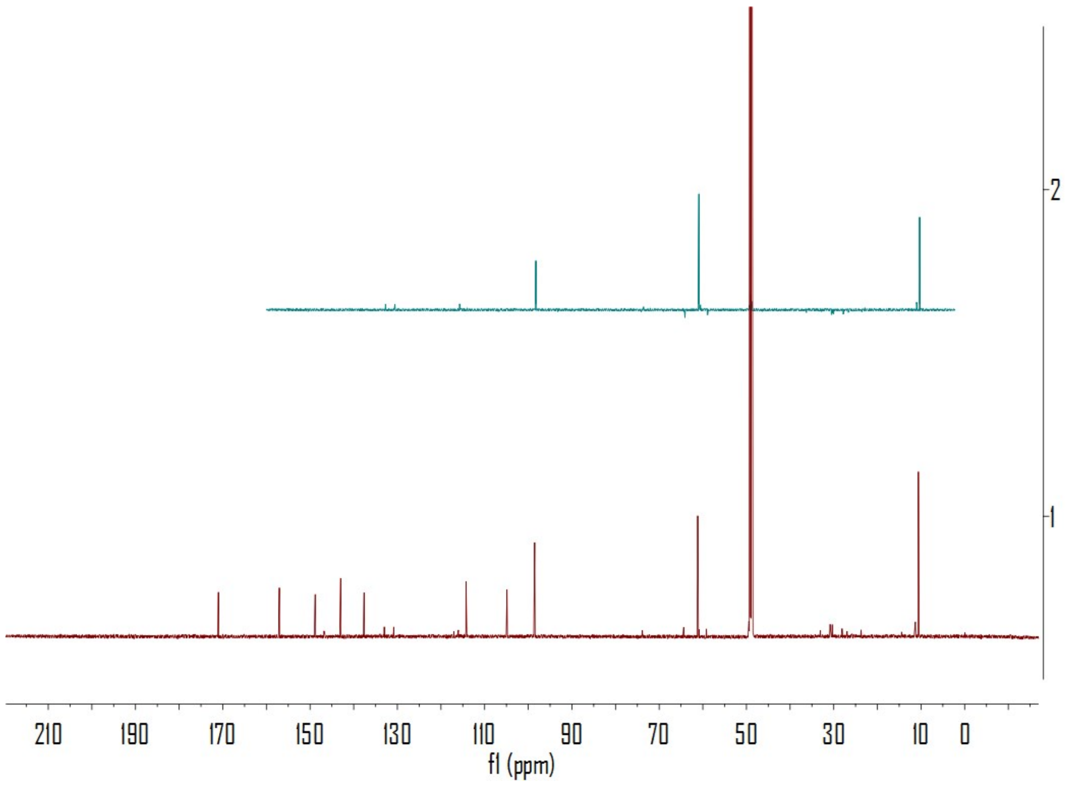

Fig.S15. DEPT 135 and ^{13}C-NMR spectrum (CD_3OD, 150MHz) of compound **(4)**

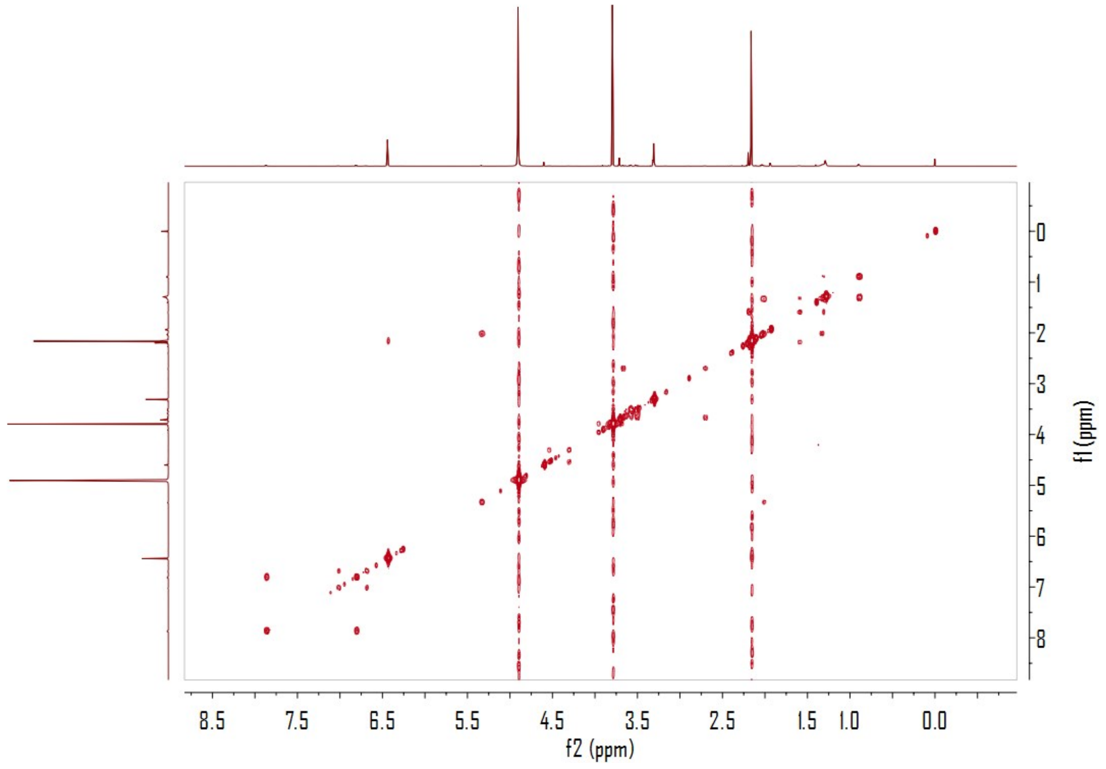

Fig.S16. ^{1}H-^{1}H COSY spectrum (CD_3OD, 600MHz) of compound **(4)**

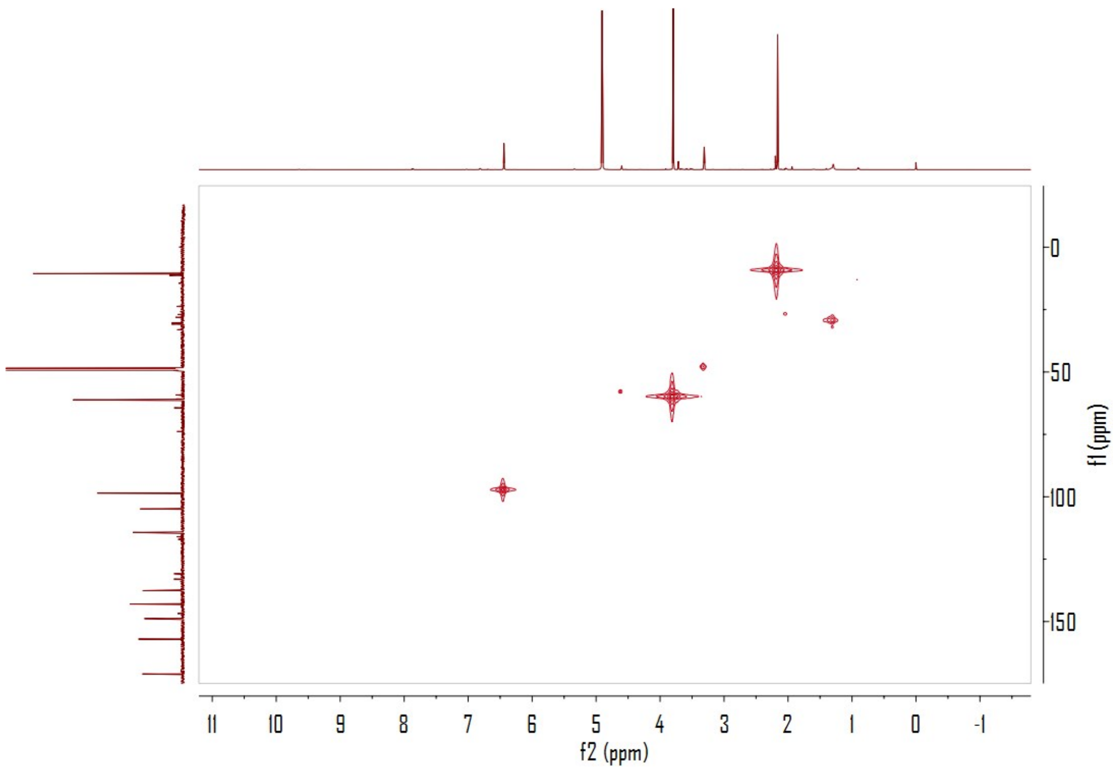

Fig.S17. HSQC spectrum (CD_3OD, 600MHz) of compound **(4)**

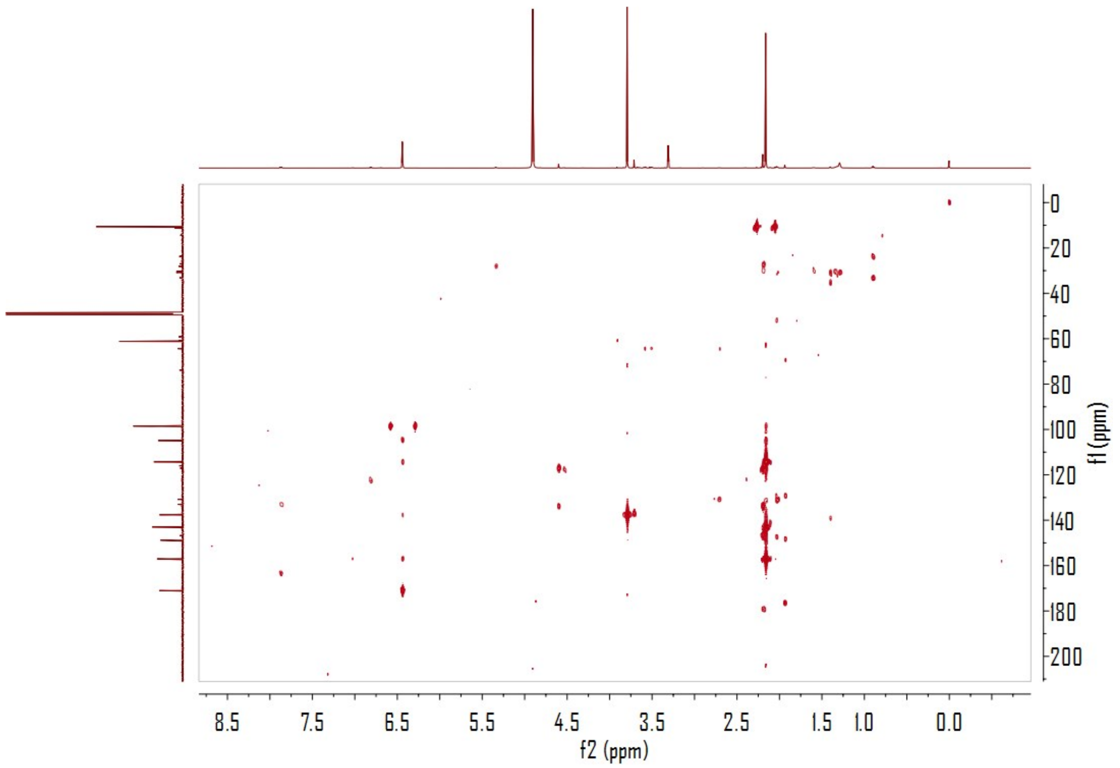

Fig.S18. HMBC spectrum (CD_3OD, 600MHz) of compound **(2)**

Table S1.deviations in NMR data between natural and synthetic[1] samples of **1**

No.	δ_C, type (N.S)	δ_C, type (S.S)	Δδ/ppm	δ_C, type (N.S)	δ_C, type (S.S)	Δδ/ppm
1	51.3, C	51.3, C	0.0			
2	66.4, CH_2	66.4, CH_2	0.0	4.21, d, (J = 10.1); 4.61, d, (J = 10.1)	4.22, d, (J = 10.1); 4.61, d, (J = 10.1)	+0.01
4	176.5, C	176.4, C	-0.1			
5	60.1, C	60.0, C	-0.1			
6	59.6, C	59.6, C	0.0			
7	65.6, CH_2	65.6, CH_2	0.0	3.74, d, (J = 10.0); 3.82, d, (J = 10.0)	3.75, d, (J = 10.0); 3.82, d, (J = 10.0)	+0.01
9	67.8, CH_2	67.8, CH_2	0.0	4.28, d, (J = 16.1); 4.38, d, (J = 16.1)	4.29, d, (J = 16.1); 4.38, d, (J = 16.1)	+0.01
10	136.9, C	136.9, C	0.0			
11	140.1, C	140.1, C	0.0			
12	190.1, C	190.9, C	+0.8			
13	69.1, CH	69.1, CH	0.0	3.08, s	3.08, s	0.0
14	88.4, C	88.4, C	0.0			
15	193.3, C	193.2, C	-0.1			
16	146.5, C	146.4, C	-0.1			
17	134.7, C	134.6, C	-0.1			
18	13.8, CH_3	13.8, CH_3	0.0	1.96, s	1.96, s	0.0
19	17.5, CH_3	17.5, CH_3	0.0	1.03, s	1.03, s	0.0
11-OH				8.97, s	8.93, s	-0.04
14-OH				6.27, s	6.24, s	+0.03
16-OH				8.84, s	8.93, s	+0.09

N.S: natural sample; S.S: synthetic sample.

Table S2. ECD Computational Result.

S2.1 Gibbs free energies and Boltzmann-population of low-energy comformers of 1*R*,5*R*,6*S*,13*R*,14*R*-**1**

Conformers of 1*R*,5*R*,6*S*,13*R*,14*R*-**1**	In MeOH	
	Δ*G*[a]	P (%)[b]
**1a**	0.0	83.3
**1b**	2.12	3.6
**1c**	2.62	5.7
**1d**	3.12	7.4

Δ*G*[a], B3LYP/ 6-31+G (d, p), in kcal/mol. [b]Boltzmann-population.

S2.2 Cartesian coordinates for the low-energy optimized conformers of 3*R*-**1** at B3LYP/6-31+G (d, p) level of theory in MeOH.

Conformer **1a**		Standard Orientation(Ångstroms)			
Center Number	Atom	Type	X	Y	Z
1	6	0	-2.241719	-0.288180	-1.025922
2	6	0	-1.814560	0.896912	-0.207483
3	6	0	-0.476924	0.740643	0.633543
4	6	0	0.191382	1.062678	-0.746580
5	6	0	0.336929	-0.354079	-1.347946
6	6	0	-1.031004	-0.932142	-1.727269

7	6	0	1.506639	1.777816	-0.753392
8	6	0	2.681302	0.868296	-0.366537
9	6	0	2.448892	-0.524237	-0.081031
10	6	0	1.038103	-1.073752	-0.137304
11	8	0	1.657428	2.975606	-1.001391
12	8	0	-3.399717	-0.665895	-1.196925
13	8	0	-1.150418	-1.857996	-2.531923
14	6	0	0.882776	-2.607136	-0.138346
15	8	0	-0.419380	-2.868273	0.517749
16	6	0	-0.877232	-1.759916	1.209259
17	6	0	0.131598	-0.612934	1.098058
18	6	0	-2.762389	1.732246	0.598435
19	8	0	-1.879635	2.596303	1.379470
20	8	0	-1.031106	1.722086	-1.243217
21	8	0	3.847180	1.376609	-0.296367
22	6	0	3.619912	-1.386728	0.266283
23	8	0	-1.933560	-1.748996	1.828067
24	6	0	0.892304	-0.545569	2.443353
25	6	0	-0.637269	1.878822	1.671805
26	1	0	0.966148	-0.389753	-2.241604
27	1	0	0.815450	-3.037213	-1.138068
28	1	0	1.651279	-3.119785	0.441758
29	1	0	-3.376921	1.088117	1.241320
30	1	0	-3.399198	2.375262	-0.008858
31	1	0	3.743664	-2.203417	-0.459599
32	1	0	4.531413	-0.787445	0.273076
33	1	0	3.500204	-1.859907	1.250797
34	1	0	1.439420	-1.477079	2.621811
35	1	0	1.609312	0.280515	2.459315
36	1	0	0.188620	-0.418523	3.269954
37	1	0	-0.693546	1.458534	2.680429
38	1	0	0.165374	2.618702	1.632425

Conformer **1b**		Standard Orientation(Ångstroms)			
Center Number	Atom	Type	X	Y	Z
1	6	0	-2.241719	-0.288180	-1.025922
2	6	0	-1.814560	0.896912	-0.207483
3	6	0	-0.476924	0.740643	0.633543
4	6	0	0.191382	1.062678	-0.746580
5	6	0	0.336929	-0.354079	-1.347946
6	6	0	-1.031004	-0.932142	-1.727269
7	6	0	1.506639	1.777816	-0.753392
8	6	0	2.681302	0.868296	-0.366537
9	6	0	2.448892	-0.524237	-0.081031
10	6	0	1.038103	-1.073752	-0.137304

11	8	0	1.657428	2.975606	-1.001391
12	8	0	-3.399717	-0.665895	-1.196925
13	8	0	-1.150418	-1.857996	-2.531923
14	6	0	0.882776	-2.607136	-0.138346
15	8	0	-0.419380	-2.868273	0.517749
16	6	0	-0.877232	-1.759916	1.209259
17	6	0	0.131598	-0.612934	1.098058
18	6	0	-2.762389	1.732246	0.598435
19	8	0	-1.879635	2.596303	1.379470
20	8	0	-1.031106	1.722086	-1.243217
21	8	0	3.847180	1.376609	-0.296367
22	6	0	3.619912	-1.386728	0.266283
23	8	0	-1.933560	-1.748996	1.828067
24	6	0	0.892304	-0.545569	2.443353
25	6	0	-0.637269	1.878822	1.671805
26	1	0	0.966148	-0.389753	-2.241604
27	1	0	0.815450	-3.037213	-1.138068
28	1	0	1.651279	-3.119785	0.441758
29	1	0	-3.376921	1.088117	1.241320
30	1	0	-3.399198	2.375262	-0.008858
31	1	0	3.743664	-2.203417	-0.459599
32	1	0	4.531413	-0.787445	0.273076
33	1	0	3.500204	-1.859907	1.250797
34	1	0	1.439420	-1.477079	2.621811
35	1	0	1.609312	0.280515	2.459315
36	1	0	0.188620	-0.418523	3.269954
37	1	0	-0.693546	1.458534	2.680429
38	1	0	0.165374	2.618702	1.632425

Conformer **1c**		Standard Orientation(Ångstroms)			
Center Number	Atom	Type	X	Y	Z
1	6	0	-2.241719	-0.288180	-1.025922
2	6	0	-1.814560	0.896912	-0.207483
3	6	0	-0.476924	0.740643	0.633543
4	6	0	0.191382	1.062678	-0.746580
5	6	0	0.336929	-0.354079	-1.347946
6	6	0	-1.031004	-0.932142	-1.727269
7	6	0	1.506639	1.777816	-0.753392
8	6	0	2.681302	0.868296	-0.366537
9	6	0	2.448892	-0.524237	-0.081031
10	6	0	1.038103	-1.073752	-0.137304
11	8	0	1.657428	2.975606	-1.001391
12	8	0	-3.399717	-0.665895	-1.196925
13	8	0	-1.150418	-1.857996	-2.531923
14	6	0	0.882776	-2.607136	-0.138346
15	8	0	-0.419380	-2.868273	0.517749

16	6	0	-0.877232	-1.759916	1.209259
17	6	0	0.131598	-0.612934	1.098058
18	6	0	-2.762389	1.732246	0.598435
19	8	0	-1.879635	2.596303	1.379470
20	8	0	-1.031106	1.722086	-1.243217
21	8	0	3.847180	1.376609	-0.296367
22	6	0	3.619912	-1.386728	0.266283
23	8	0	-1.933560	-1.748996	1.828067
24	6	0	0.892304	-0.545569	2.443353
25	6	0	-0.637269	1.878822	1.671805
26	1	0	0.966148	-0.389753	-2.241604
27	1	0	0.815450	-3.037213	-1.138068
28	1	0	1.651279	-3.119785	0.441758
29	1	0	-3.376921	1.088117	1.241320
30	1	0	-3.399198	2.375262	-0.008858
31	1	0	3.743664	-2.203417	-0.459599
32	1	0	4.531413	-0.787445	0.273076
33	1	0	3.500204	-1.859907	1.250797
34	1	0	1.439420	-1.477079	2.621811
35	1	0	1.609312	0.280515	2.459315
36	1	0	0.188620	-0.418523	3.269954
37	1	0	-0.693546	1.458534	2.680429
38	1	0	0.165374	2.618702	1.632425

Conformer **1d**		Standard Orientation(Ångstroms)			
Center Number	Atom	Type	X	Y	Z
1	6	0	-2.241719	-0.288180	-1.025922
2	6	0	-1.814560	0.896912	-0.207483
3	6	0	-0.476924	0.740643	0.633543
4	6	0	0.191382	1.062678	-0.746580
5	6	0	0.336929	-0.354079	-1.347946
6	6	0	-1.031004	-0.932142	-1.727269
7	6	0	1.506639	1.777816	-0.753392
8	6	0	2.681302	0.868296	-0.366537
9	6	0	2.448892	-0.524237	-0.081031
10	6	0	1.038103	-1.073752	-0.137304
11	8	0	1.657428	2.975606	-1.001391
12	8	0	-3.399717	-0.665895	-1.196925
13	8	0	-1.150418	-1.857996	-2.531923
14	6	0	0.882776	-2.607136	-0.138346
15	8	0	-0.419380	-2.868273	0.517749
16	6	0	-0.877232	-1.759916	1.209259
17	6	0	0.131598	-0.612934	1.098058
18	6	0	-2.762389	1.732246	0.598435
19	8	0	-1.879635	2.596303	1.379470

20	8	0	-1.031106	1.722086	-1.243217
21	8	0	3.847180	1.376609	-0.296367
22	6	0	3.619912	-1.386728	0.266283
23	8	0	-1.933560	-1.748996	1.828067
24	6	0	0.892304	-0.545569	2.443353
25	6	0	-0.637269	1.878822	1.671805
26	1	0	0.966148	-0.389753	-2.241604
27	1	0	0.815450	-3.037213	-1.138068
28	1	0	1.651279	-3.119785	0.441758
29	1	0	-3.376921	1.088117	1.241320
30	1	0	-3.399198	2.375262	-0.008858
31	1	0	3.743664	-2.203417	-0.459599
32	1	0	4.531413	-0.787445	0.273076
33	1	0	3.500204	-1.859907	1.250797
34	1	0	1.439420	-1.477079	2.621811
35	1	0	1.609312	0.280515	2.459315
36	1	0	0.188620	-0.418523	3.269954
37	1	0	-0.693546	1.458534	2.680429
38	1	0	0.165374	2.618702	1.632425

References

[1] Ellerbrock, P.; Armanino, N.; Ilg, M. K.; Webster, R.; & Trauner, D. An eight-step synthesis of Epicolactone reveals its biosynthetic origin. *Nature Chemistry*. **2015**, 7(11), 879-882.
